# A population-based study on trajectories of HER2 status during neoadjuvant chemotherapy for early breast cancer and metastatic progression

**DOI:** 10.1038/s41416-024-02777-6

**Published:** 2024-06-28

**Authors:** Caroline Boman, Xingrong Liu, Louise Eriksson Bergman, Wenwen Sun, Christian Tranchell, Maria Angeliki Toli, Balazs Acs, Jonas Bergh, Theodoros Foukakis, Alexios Matikas

**Affiliations:** 1 Karolinska Institutet, Oncology/Pathology Department, Stockholm, Sweden; 2 Breast Center, Karolinska Comprehensive Cancer Center, Stockholm, Sweden; 3 Department of Surgery and Oncology, Capio Sankt Göran Hospital, Stockholm, Sweden; 4 Department of Clinical Pathology and Cancer Diagnostics, Karolinska University Hospital, Stockholm, Sweden

**Keywords:** breast cancer, HER2 change, HER2-low, neoadjuvant chemotherapy, prognosis, Breast cancer, Breast cancer

## Abstract

**Background:**

This study aimed to investigate the distribution and changes of HER2 status in untreated tumours, in residual disease and in metastasis, and their long-term prognostic implications.

**Methods:**

This is a population-based cohort study of patients treated with neoadjuvant chemotherapy for breast cancer during 2007–2020 in the Stockholm–Gotland region which comprises 25% of the entire Swedish population. Information was extracted from the National Breast Cancer Registry and electronic patient charts to minimize data missingness and misclassification.

**Results:**

In total, 2494 patients received neoadjuvant chemotherapy, of which 2309 had available pretreatment HER2 status. Discordance rates were 29.9% between primary and residual disease (kappa = 0.534), 31.2% between primary tumour and metastasis (kappa = 0.512) and 33.3% between residual disease to metastasis (kappa = 0.483). Adjusted survival curves differed between primary HER2 0 and HER2-low disease (*p* < 0.001), with the former exhibiting an early peak in risk for death which eventually declined below the risk of HER2-low. Across all disease settings, increasing the number of biopsies increased the likelihood of detecting HER2-low status.

**Conclusion:**

HER2 status changes during neoadjuvant chemotherapy and metastatic progression, and the long-term behaviours of HER2 0 and HER2-low disease differ, underscoring the need for obtaining tissue biopsies and for extended follow-up in breast cancer studies.

## Introduction

Historically, the expression of two prognostic and predictive markers, oestrogen receptor (ER) and human epidermal growth factor receptor 2 (HER2), has classified breast cancer (BC) into three distinct, non-overlapping subtypes: ER-positive/HER2-negative, HER2-positive and triple negative. This classification is easy to implement, reproducible and has clinical utility in terms of both prognostication and selection of appropriate treatment. Notwithstanding geographical and temporal variations in commonly accepted positivity cut-offs of ER and HER2 [1] and the development of molecular subtyping [2], the dominance of immunohistochemistry (IHC)-based subtyping had remained undisputed.

Despite the overwhelming success and widespread acceptance of clinical subtyping, it is now challenged not due to a deepening understanding of the underlying biology, but rather to the emergence of HER2-low expression as an important predictive biomarker. Following the results of the DESTINY-Breast04 trial, trastuzumab deruxtecan gained regulatory approval for the treatment of metastatic BC with low HER2 expression since it prolonged overall survival compared to physician’s choice chemotherapy [3]. These results sparked an interest in HER2-low BC, with numerous studies reporting on its prognosis [4], analytical difficulties [5], change of HER2 status [6], and biology of HER2-low disease at the genomic and transcriptomic level [7]. The cumulative evidence indicates that HER2-low BC is not a separate disease entity, although this conclusion does not diminish its relevance as a clinically pertinent marker expressed in approximately half of patients with metastatic BC.

Most studies reporting on HER2-low BC have been retrospective single- or multi-institutional cohort studies thus susceptible to selection bias or based on registries known for underreporting of treatment data [8]. With the present population-based study performed during a time period when contemporary therapies had been introduced to clinical practice, with near total completeness thanks to cross-checking of patient charts and long-term follow-up, we aimed to investigate the dynamic changes of HER2 status during neoadjuvant therapy (NAT) and metastatic progression, and their prognostic implications.

## Methods

### Study design

This is a retrospective, population-based, analytical, prognostic cohort study of patients treated with neoadjuvant chemotherapy for non-metastatic BC. The primary objective of this study is to investigate the distribution and dynamic changes of HER2 status as defined hereunder, in primary tumours, in residual disease following neoadjuvant chemotherapy and in metastasis, including the incidence of HER2 0, HER2-low and HER2-positive disease per metastatic site and according to the number of obtained biopsies. The secondary objective is to investigate the prognostic implications of HER2 status at baseline, residual disease, and metastasis in terms of short-term and long-term patient outcomes. This study was approved by the ethics review committee in Stockholm (2016/1303-31 with amendments 2018/1049-32, 2021-01147 and 2023-02918-02). The ethical approval granted a waiver for informed consent in this non-interventional collection and analysis of data from registries and patient records. The reporting of this study follows the STROBE (Strengthening the Reporting of Observational Studies in Epidemiology) guidelines [9] and the European Society for Medical Oncology Guidance for Reporting Oncology real-World Evidence (ESMO-GROW) checklist [10] which is provided as supplementary data.

### Data source and patient cohort

All individuals diagnosed with invasive BC diagnosed from January 1, 2007 to December 31, 2020 in the region of Stockholm–Gotland, Sweden, were identified through the National Breast Cancer Register (NBCR). The Stockholm–Gotland region represents approximately 25% of the entire Swedish population. The NBCR is prospectively maintained and has high coverage and completeness of 99.9% [11], using the Swedish Cancer Register as a reference to which reporting of all cancer cases is mandatory by law. By using the unique ten-digit Personal Identity Number assigned to all persons registered in Sweden, the NBCR was linked to the electronic patient charts in order to minimize data missingness and misclassification. Extracted information from patient charts included tumour characteristics at baseline, residual disease, and metastasis such as hormone receptor expression, HER2 expression and gene amplification, grade, Ki67, number of removed and positive lymph nodes, tumour size and site of metastasis. In addition, details on administered treatment and follow-up status, including the date and site of first relapse, were extracted.

**Fig. 1:**
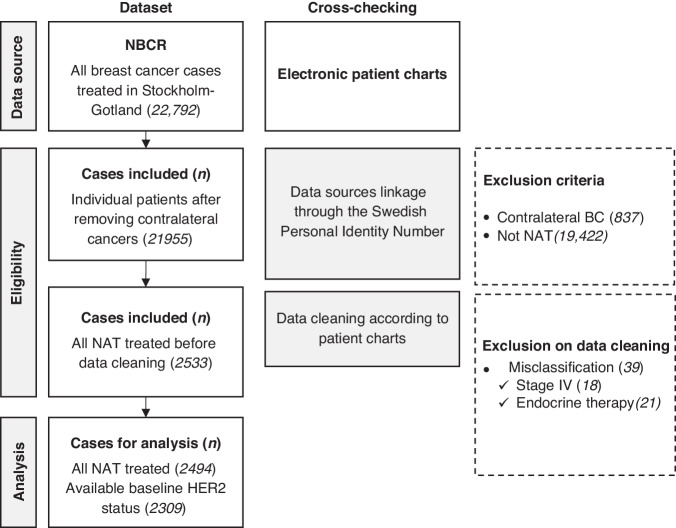
Flowchart of patient selection, reported according to the European Society for Medical Oncology Guidance for Reporting Oncology Real-World Evidence (ESMO-GROW). NBCR National Breast Cancer Register, NAT neoadjuvant treatment, HER2 human epidermal growth factor receptor 2.

The study cohort comprises all patients diagnosed with non-metastatic invasive BC (stage I–III, defined as no metastatic disease identified within three months from BC diagnosis) that were treated with chemotherapy as first treatment, regardless of eventual breast surgery following NAT. Patients receiving endocrine therapy only were excluded, since it was mostly used as primary treatment in patients with advanced age and comorbidities, without intention to proceed to surgery. Patients with synchronous bilateral BC, defined as diagnosis of BC in the contralateral breast within three months from BC diagnosis, were excluded. The patient flowchart is shown in Fig. 1.

Information on HER2 protein expression according to immunohistochemistry (IHC 0 to 3 + ) and gene amplification according to in situ hybridization (ISH, *ERBB2* gene copy number and ratio of *ERBB2* gene copies to chromosome centromere 17 copies) was extracted. HER2 status assessment followed the 2018 American Society of Clinical Oncology/College of American Pathologists guidelines: HER2 positive was defined as HER2 IHC 3 + , or 2+ and positive ISH ( ≥ 6.0 *ERBB2* copies, or 4.0–6.0 copies and ratio  ≥ 2.0); HER2 low was defined as HER2 IHC 1 + , or 2+ and negative ISH; finally, HER2 0 was defined as HER2 IHC 0 [1, 12]. According to Swedish national guidelines, positivity cut-offs for oestrogen and progesterone receptor expression according to IHC are defined as  ≥ 10%. ER-positive/HER2-negative BC subtypes were determined in accordance with the St Gallen classification [13]: Luminal A-like was defined as progesterone receptor (PR) ≥ 20% and Ki67 < 15%, whereas Luminal B-like was defined as PR < 20% or Ki67 ≥ 15%. Pathology data were extracted from pathology reports and no rescoring of ER, PR and HER2 was performed, except for patients described below.

### Rescoring of HER2 status

For randomly selected patients treated between 2016 and 2018, HER2 status was reassessed on untreated baseline archival tissue by a trained pathologist blinded to the original assessment (W.S.). Formalin-fixed paraffin-embedded material was stained with PATHWAY anti-Her2/neu (4B5) rabbit monoclonal primary antibody (Roche Diagnostics International, Rotkreutz, Switzeland) as described by the manufacturer (BenchMark ULTRA IHC/ISH Staining Module, Ventana Medical Systems, Inc., Arizona, USA). HER2 IHC scoring was performed in line with the ASCO/CAP 2018 guidelines as follows [1]: specimens with no detectable HER2 staining were defined as HER2 0; specimens with faint membrane staining not fulfilling criteria for 1+ were defined as HER2 “ultra-low”; 1+ was defined as incomplete faint/weak membrane staining in > 10%; 2+ as weak to moderate complete membrane staining in > 10%, or complete intense staining in ≤ 10%; 3+ as complete intense membrane staining in > 10% of tumour cells.

### Endpoints

Within the scope of this study, both pathologic response to treatment and time-to-event endpoints follow the standardized definitions for efficacy endpoints in neoadjuvant breast cancer trials (NeoSTEEP) [14]. Pathologic complete response (pCR) was defined as an absence of invasive tumour in the breast and the axilla, while in situ carcinoma was allowed (ypT0/Tis and ypN0). Patients that initiated NAT but were not operated, regardless of reason (disease progression, death prior to surgery, patient’s wish), were regarded as non-pCR. Breast cancer-free survival (BCFS) was defined as time from surgery to occurrence of one of the following events: local, locoregional or distant relapse, contralateral BC, or death due to any cause. Distant relapse-free survival (DRFS) was defined as time from surgery to distant relapse or death due to any cause, whichever occurred first. Finally, overall survival (OS) was defined as time from BC diagnosis to death due to any cause. Patients were censored if they were lost to follow-up due to migration or did not have the event of interest when the study observation period ended (May 2, 2023).

### Statistical analysis

The distribution of patient and disease characteristics at diagnosis was calculated and classified by primary HER2 status (HER2 0, HER2-low, and HER2-positive). For continuous variables, the median and interquartile range were reported and distribution differences across groups were compared using the Kruskal–Wallis test, whereas frequencies and percentages of categorical variables were compared using the Chi-square test or Fisher’s exact test.

Based on available HER2 data, distributions of HER2 status were summarized for each disease setting (primary disease, residual disease, and metastasis) separately, stratified by ER status, and further presented according to metastatic sites, ER-status, ER-expression category, and St Gallen subtypes. The likelihood of patients having at least one HER2-low biopsy was calculated based on the total number of biopsies across the entire disease trajectory, from primary to residual to metastatic disease. Cohen’s kappa coefficient was used to evaluate the HER2 concordance rates [15]. Dynamic change of HER2 status and discordance between primary disease, residual disease, and metastasis were graphically presented using Sankey diagrams and pie-of-pie charts, respectively.

Kaplan–Meier curves and adjusted survival probabilities using standardization were estimated to compare outcomes of patients according to HER2 status per disease setting and HER2 status change between settings [16, 17]. When proportional hazard assumptions were violated, flexible parametric survival models were applied to estimate adjusted (or standardized) survival probabilities for each group while accounting for other prognostic factors (age, ER expression, T stage, nodal status, grade, Ki67 and type of neoadjuvant chemotherapy), and non-proportional hazards for HER2 status were estimated using natural cubic splines with two degrees of freedom [18, 19]. In the neoadjuvant chemotherapy setting pCR is an intermediate outcome of interest based on its correlation with survival and was thus explored as a mediator in the pathway between primary HER2 status and overall survival in a mediation analysis. Additionally, to address the potential impact of missing data and to validate our findings, sensitivity analyses were conducted using flexible parametric models on the complete dataset (excluding patients with missing data) and multiple imputed datasets through multiple imputation approaches [20–22].

Data management and statistical analysis were performed using SAS software (version 9.4; SAS Institute, Cary, NC) and R (version 4.3.1, June 2023). A two-sided P-value of less than 0.05 was considered to be statistically significant. The code used to generate results of this article can be accessed upon reasonable request to the corresponding author.

## Results

### Patient characteristics

In total, 2494 patients were treated with neoadjuvant chemotherapy, of which 2309 (92.5%) had available pretreatment HER2 status. The median follow-up time was 6.36 years (interquartile range, 4.29–9.54 years). The distribution of patients’ clinical and pathological characteristics according to prechemotherapy HER2 status is shown in Table 1. HER2-low and HER2 0 cancers were ER-positive in 78.3% and 52.0% of cases, respectively (Chi-square *p* < 0.001). When stratifying according to ER status, patient characteristics were mostly balanced between HER2-low and HER2 0, with the observed between-group differences attributed mainly to HER2-positive tumours (Supplementary Tables 1, 2).

**Table 1:** Distribution of patient characteristics according to prechemotherapy HER2 status in the total population.

	HER2 0 N (%)	HER2-low N (%)	HER2-positive N (%)	*P*-value *
Patients	642	847	820	
Median age (IQR)	50.0 (42.7–59.6)	51.6 (43.8–62.5)	52.3 (43.5–61.6)	0.032
Chemotherapy				<0.001
Antracycline and taxane	552 (86.0)	688 (81.2)	607 (74.0)	
Antracycline	40 (6.2)	44 (5.2)	8 (1.0)	
Taxane	50 (7.8)	113 (13.3)	145 (17.7)	
Other	0 (0.0)	2 (0.2)	60 (7.3)	
T stage				0.144
T0-1	110 (17.2)	134 (15.9)	119 (14.5)	
T2	384 (60.0)	469 (55.6)	500 (61.1)	
T3	121 (18.9)	204 (24.2)	167 (20.4)	
T4	25 (3.9)	37 (4.4)	32 (3.9)	
Missing	2	3	2	
Nodal status				0.056
Negative	307 (48.3)	356 (42.2)	377 (46.0)	
Positive	329 (51.7)	488 (57.8)	442 (54.0)	
Missing	6	3	1	
Histologic type				<0.001
Ductal	475 (77.7)	641 (79.5)	689 (89.4)	
Lobular	46 (7.5)	60 (7.4)	16 (2.1)	
Other	90 (14.7)	105 (13.0)	66 (8.6)	
Missing	31	41	49	
Grade				<0.001
Grade 1	15 (2.7)	28 (3.6)	5 (0.7)	
Grade 2	211 (37.5)	395 (51.3)	283 (38.5)	
Grade 3	337 (59.9)	347 (45.1)	447 (60.8)	
Missing	79	77	85	
Oestrogen receptor				<0.001
Negative	308 (48.0)	184 (21.7)	326 (39.9)	
Positive	334 (52.0)	662 (78.3)	491 (60.1)	
Missing	0	1	3	
Progesterone receptor				<0.001
Negative	397 (61.8)	352 (41.7)	480 (58.8)	
Positive	245 (38.2)	493 (58.3)	337 (41.2)	
Missing	0	2	3	
Ki67				<0.001
Median (IQR)	50 (30–75)	37 (25–60)	40 (30–60)	
Missing	6	25	20	
Year of diagnosis				0.067
2007–2010	96 (15.0)	146 (17.2)	104 (12.7)	
2011–2014	137 (21.3)	182 (21.5)	171 (20.9)	
2015–2018	243 (37.9)	307 (36.2)	352 (42.9)	
2019–2020	166 (25.9)	212 (25.0)	193 (23.5)	

*Kruskal–Wallis tests for continuous variables (age, Ki67); Chi-square tests used for all others.

*IQR* interquartile range.

### HER2 status in primary, residual and metastatic disease

Rates of HER2-positive, HER2-low and HER2 0 BC in all available unmatched samples and stratified according to ER status, at primary disease, residual disease following NAT and at metastatic biopsy, as well as for patients with biopsies from all three timepoints (primary, residual and metastatic disease), are shown in Supplementary Fig. 1. The proportion of HER2-low was relatively stable at the different disease settings and was higher in the ER-positive compared to the ER-negative group (from 45 to 52% and from 22 to 28%). Interestingly, while the proportion of HER2-negative tumours was stable across different levels of ER expression, the relative proportions of HER2 0 and HER2-low BC varied substantially: 38% versus 23% respectively in ER 0%, 32% versus 21% in ER 1–9%, reaching 21% versus 49% respectively in ER ≥ 95% (Cochran-Armitage *p* < 0.001; Supplementary Fig. 2). Finally, while HER2 0 status was mostly associated with ER negativity, numerically more HER2-negative tumours were classified as HER2 0 within the Luminal A-like than within the Luminal B-like subtype: 41% versus 33%, respectively (*p* = 0.12).

The probability of detecting HER2-low status increased with the number of available biopsies at any setting, in both the ER-positive and ER-negative subgroups, as shown in Supplementary Fig. 3. In the ER-positive subgroup, the probability of at least one biopsy indicating HER2-low status increased from 35.3% in patients with one biopsy to 66.7% in those with two biopsies, while the corresponding proportions in the ER-negative group were 19.0% and 40.8%, respectively.

### Rescoring of HER2 status in prechemotherapy and residual disease samples

In total, 107 HER2 0 and HER2-low baseline samples were rescored, of which 70 were HER2 1+ or HER2 2+ without gene amplification, and 37 were HER2 0 according to the original assessment. At rescoring, all 70 HER2 1 + /2+ cases were assessed as HER2-low BC. Of the 37 HER2 0 samples, 36 were assessed as HER2 “ultra-low” and one as HER2 0. In addition, 9 HER2 0 and 22 HER2-low residual disease samples were rescored. All HER2 1 + /2+ cases were assessed as HER2-low BC, whereas of the 9 HER2 0 samples, 7 were assessed as HER2 “ultra-low” and two as HER2 0.

### Change of HER2 status between primary and residual disease

**Fig. 2:**
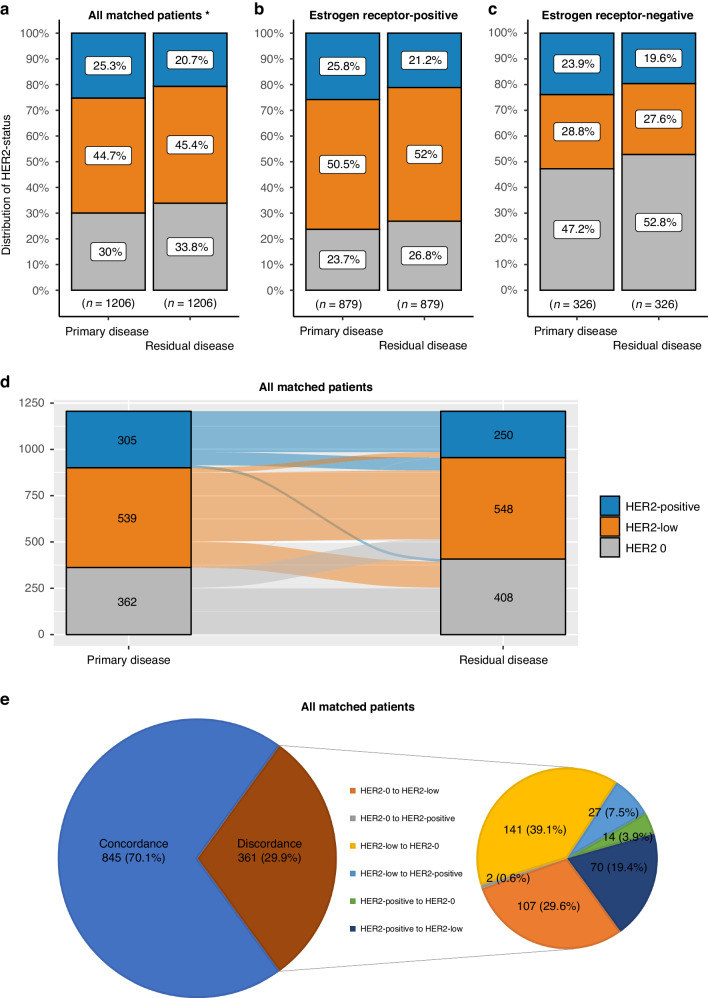
Distribution of HER2 status at baseline and residual disease. Distribution of HER2-positive, HER2-low and HER2 0 in matched samples at baseline and residual disease (**a**), in oestrogen receptor positive samples (**b**) and in oestrogen receptor negative samples (**c**). Sankey diagram describing changes of HER2 status from primary to residual disease (**d**). Pie-of-pie chart describing discordance of HER2 status between primary and residual disease (**e**).

The change of HER2 status from primary to residual disease following NAT in matched samples with available information is depicted in Fig. 2. While the proportions of the three HER2 states were similar between primary and residual disease in the entire population and according to ER status, discordance was substantial and was observed in 29.9% of all cases (Cohen’s kappa = 0.534, *p* < 0.001). The majority of discordant cases concerned change from HER2 0 to HER2-low, or vice versa.

Since missingness of HER2 status at residual disease is informative, we repeated this analysis on all patients regardless of available information. These results are shown in Supplementary Fig. 4. Less than half (43.8%) of all HER2-low BC remained HER2-low at residual disease, whereas similar proportions (14.4% and 16.6%) achieved pCR and changed to HER2 0, respectively. Change from HER2-low to HER2-positive BC was uncommon (3.2%). Regarding baseline HER2-positive tumours, pCR was achieved in 45.6% of all cases, while HER2 loss (change to HER2 0 or HER2-low) was observed in 10.4% of patients with residual disease.

### Clinical outcomes according to HER2 status

Consistent with previous reports, pCR rates were higher in HER2 0 than in HER2-low patients (20.5% versus 14.4%, *p* = 0.003). However, this difference was no longer observed after stratification by ER status (ER-negative: 32.1% versus 35.9%, *p* = 0.45 and ER-positive: 9.6% versus 8.5%, *p* = 0.62). HER2-positive tumours, 95.4% of which were treated with HER2-targeting agents preoperatively, had the highest probability of achieving pCR regardless of ER status: 45.6% in the entire population and 60.7% and 35.2% in the ER-negative and ER-positive subgroups, respectively. Within both the HER2 0 and the HER2-low groups, patients treated with both anthracyclines and taxanes preoperatively had the highest probability of attaining pCR compared to anthracyclines only or taxanes only, though wide confidence intervals were observed in multivariable logistic regression models (Supplementary Table 3).

In the entire population and in subgroups defined by ER status, the Kaplan–Meier curves for OS of HER2-low and HER2 0 BC crossed, implying violation of proportionality of hazards (logrank *p* < 0.001; Supplementary Fig. 5). Moreover, unadjusted Kaplan–Meier estimates of OS showed no difference between HER2 0 and HER2-low in Luminal A-like (logrank *p* = 0.35) and Luminal B-like tumours (logrank *p* = 0.81).

**Fig. 3:**
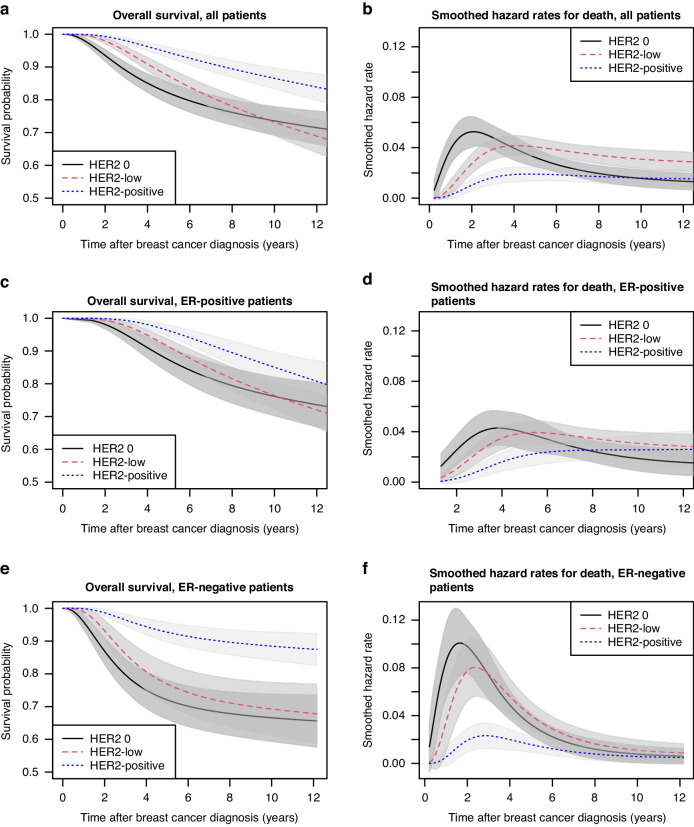
Overall survival according to baseline HER2 status. Standardized survival probability curves according to baseline HER2 status, adjusted for age, type of chemotherapy, nodal status, T stage, grade, oestrogen receptor status and Ki67 in total (**a**) oestrogen receptor positive (**c**) and estrogen receptor negative (**e**) populations. Smoothed hazards plots for death according to baseline HER2 status in total (**b**), oestrogen receptor positive (**d**) and oestrogen receptor negative (**f**) populations.

We thus calculated standardized survival probabilities depending on HER2 status at primary disease in the entire population and by ER status and compared survival between the different groups using multivariable flexible parametric survival models. HER2-positive tumours were associated with improved overall survival at 10 years regardless of ER status, with ten-year rates of 86.5% (95% Confidence Interval [CI] 83.3–89.8%). Mediation analysis indicated that only 36% (95% CI 21–51%) of this improvement in OS could be attributed to the increased pCR rates, after adjusting for age, type of treatment, ER status, T stage, grade and Ki67. In the comparison of HER2 0 and HER2-low BC, OS differed in adjusted models (likelihood ratio test *p* < 0.001) although the standardized ten-year survival probabilities were similar: 73.1% (95% CI 69.1–77.2%) for HER2 low and 73.6% (95% CI 69.2–77.9%) for HER2 0 disease. When plotting hazards over time, the observed peak at the two-year time point was less pronounced in HER2-low than in HER2 0 disease regardless of ER status and eventually surpassed that of HER2 0 BC, findings that imply different disease behaviour even within seemingly homogeneous subgroups defined by ER expression. These results are summarized in Fig. 3. Similar findings were noted for the BCFS and DRFS endpoints, with HER2 status defined at primary (Supplementary Fig. 6) or at residual disease (Supplementary Fig. 7).

There was no association between the change of HER2 status from primary to residual disease from positive to negative (HER2 loss) or vice versa (HER2 gain) and BCFS, DRFS or OS (Supplementary Fig. 8). However, the number of patients in these comparisons was small.

### Sensitivity analysis

Since missingness in retrospective breast cancer studies has been associated with worse outcomes [21], we performed multiple imputation of all missing data at primary disease based on 500 imputed datasets. Compared to the multiple imputation approach, the complete case analysis of all patients with available information slightly overestimated overall survival in all patient groups defined by HER2 status, but the difference in adjusted OS probability between HER2 0 and HER2-low remained (likelihood ratio test p < 0.001; Supplementary Fig. 9).

### Evolution of HER2 status during metastatic progression

**Fig. 4:**
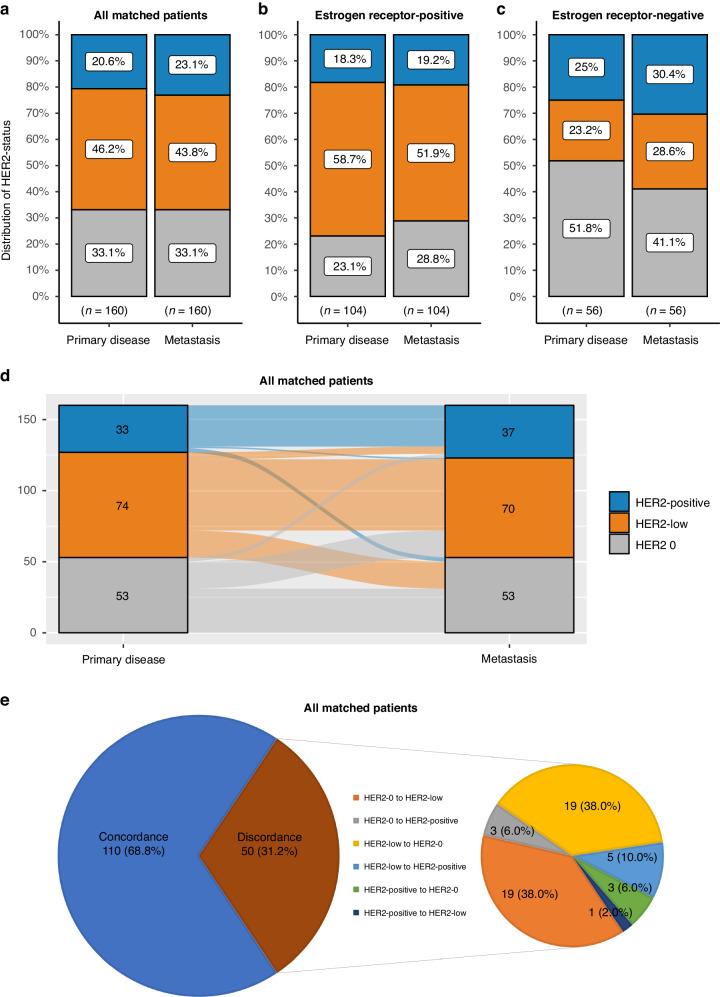
Distribution of HER2 status at baseline and metastasis. Distribution of HER2-positive, HER2-low and HER2 0 in matched samples at baseline and metastasis (**a**), in oestrogen receptor positive samples (**b**) and in oestrogen receptor negative samples (**c**). Sankey diagram describing changes of HER2 status from primary to metastatic disease (**d**). Pie-of-pie chart describing discordance of HER2 status between primary and metastatic disease (**e**).

**Fig. 5:**
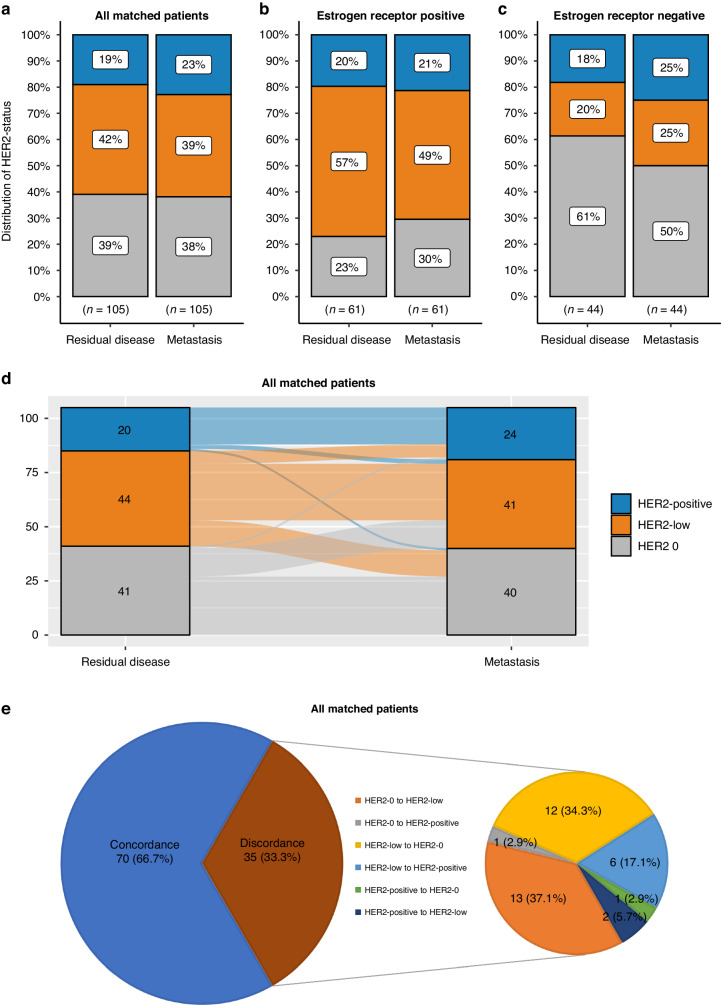
Distribution of HER2 status at residual disease and metastasis. Distribution of HER2-positive, HER2-low and HER2 0 in matched samples at residual disease and metastasis (**a**), in oestrogen receptor positive samples (**b**) and in oestrogen receptor negative samples (**c**). Sankey diagram describing changes of HER2 status from residual to metastatic disease (**d**). Pie-of-pie chart describing discordance of HER2 status between residual and metastatic disease (**e**).

The rates and change of HER2 status from primary and residual to metastatic disease is shown in Figs. 4, 5. Although rates of HER2 0 and HER2-low disease were similar between primary/residual and metastatic disease, discordance was observed in approximately a third of all matched samples (primary to metastatic disease Cohen’s kappa = 0.512, *p* < 0.001 and residual to metastatic disease Cohen’s kappa = 0.483, *p* < 0.001).

Significant variation in the relative proportions of HER2 status (0, low and positive) was observed according to the site of metastasis. HER2 0 was mostly observed in lung (40% of all lung biopsies) and bone metastases (50%), HER2-low in liver metastases (53%) and HER2-positive status in central nervous system metastases (48%). Same patterns were noted when stratifying according to ER status, although the number of patients in these analyses was small (Supplementary Fig. 10). In addition, HER2 low status was detected in 41.1% of patients with one and 45.0% of patients with two metastatic biopsies. The concordance of HER2 status between the first and second metastatic biopsy was moderate (Cohen’s kappa = 0.486).

The unadjusted Kaplan–Meier estimates of OS and the adjusted survival probabilities according to HER2 status at metastasis are shown in Supplementary Fig. 11. Median OS since time of distant recurrence in the entire cohort was 15.0 months (95% CI 13.7–17.4), and the difference in adjusted survival between HER2-low and HER2 0 disease at metastasis did not differ statistically significantly (*p* = 0.169). No patients were treated with trastuzumab deruxtecan.

## Discussion

In this retrospective analysis of a prospectively collected population-based cohort, we investigate the trajectories and clinical correlates of HER2 status before neoadjuvant chemotherapy, at residual invasive disease and at breast cancer metastasis. While other studies have reported on the changes in HER2 status during preoperative treatment, with this analysis we confirm prior results and contribute to the literature with new findings on the long-term behaviour of HER2-low disease and the effect of a number of biopsies and metastatic site on the likelihood of detecting HER2-low status. Concerning the clinically relevant HER2-low status, we observed it in approximately 36–45% of cases across the various disease settings, strongly dependent on ER expression. Change of HER2-low status between settings was seen in almost one third of all cases. pCR was achieved by 14.4% of patients, while 10-year survival rates were 73.1%.

HER2 0 and HER2-low breast cancer are consistently associated with ER-negative and ER-positive status respectively, which we confirmed regardless of disease setting. In addition, we observed in “ER-low” tumours a distribution of HER2 status resembling that of ER-negative breast cancer, thus adding another line of evidence on the ambiguous nature of “ER-low” breast cancer that likely represents ER-negativity based on molecular and clinical data [23–26]. Furthermore, we found discordance rates of 29.9% between primary and residual disease, similar to most [27–29], but not all studies [30]. Crucially, we found that increasing number of biopsies sharply increased the likelihood of at least one showing HER2-low status. This observation expanded to the metastatic setting, where HER2 status showed additional tropism to specific metastatic sites as also highlighted by a small post-mortem study [31]. Considering the importance of HER2-low as a predictive biomarker and its substantial temporal and spatial heterogeneity every effort should be made in routine practice to obtain tumour tissue at multiple timepoints, a lesson previously learned from the similar example of ER expression [32, 33]. The recently presented results of the DESTINY-Breast06 trial showed that the relative and absolute benefit from trastuzumab deruxtecan is the same between metastatic HER2-low and “ultra-low” cancer [34], which expands the candidate population and further underscores the importance of our findings and conclusions, especially concerning the proportion of “ultra-low” assessments among our rescored HER2 0 biopsies. Nevertheless, the proportion of “ultra-low” cases in our study was substantially higher than previously reported at the advanced disease setting (93% versus 60%) [35]. However, the lack of standardized guidance on rescoring and optimized assays that distinguish low expression levels, and small sample size means that our results should not be overinterpreted.

The association of HER2 status and long-term outcomes remains uncertain. An initial study showed improved survival of patients with HER2-low compared to HER2 0 disease regardless of ER status [36], while another showed no difference when stratifying by ER status [37]. Larger studies [38] and a trial-level meta-analysis [4] have since then shown that even when stratifying by ER status, HER2-low breast cancer is associated with slightly improved survival. However, previous studies have either had short median follow-up or had not evaluated whether the proportionality of hazards assumption holds [27, 30, 36]. Here, we demonstrate that HER2 0 disease, regardless of ER status, is associated with an early increase in risk for death which eventually decreases below the risk for death of HER2-low patients. This observation was not previously captured which underscores the need to pursue long-term patient follow-up, considering the long natural history of non-metastatic breast cancer [39].

The reasons that explain the clinical behaviour of HER2-low disease are unclear. Subtle differences between HER2-low and HER2 0 in terms of *TP53* mutations and gene expression have been previously reported and indicate an overall “luminal-like” pattern for HER2-low BC [7, 36, 40]. In addition, HER2-driven tumour cell dormancy has been previously demonstrated [41, 42] and might explain the long-term natural history of HER2-low BC in light of higher *ERBB2* expression compared to HER2 0 BC [7] and lack of prolonged adjuvant therapy as in HER2-positive BC, that might eradicate or at least maintain dormancy [42]. In addition, we demonstrate that the repeatedly reported improved survival of HER2-positive breast cancer cannot be explained only based on increased pCR rates compared to the HER2-negative subgroups. Whether this phenomenon can be attributed to a shift of the non-responding population away from the poor prognosis Residual Cancer Burden III class [27], to the eradication of minimal residual disease by prolonged adjuvant HER2-blockade, or to other factors is unclear.

Strengths of our study include its size, the population-based design that limits selection bias compared to studies of consecutive patients from single or few sites, and the cross-checking with patient charts that decreased missingness and misclassification compared to registry-based studies. In addition, we didn’t limit our focus on the comparison between pre- and post-chemotherapy HER2 status but expanded even to metastatic progression and went beyond simple descriptive analyses and crude associations of HER2 status with outcomes to study the behaviour of HER2-low disease with a significantly longer follow-up time than previous studies. Missing information on HER2 status from prechemotherapy biopsies was limited and sensitivity analysis with multiple imputations showed very little difference with the observed outcomes. Missingness from residual disease was due to the now-abandoned practice of not retesting residual cancer for HER2 status. Regarding metastatic biopsies, these are routinely obtained in clinical practice and in previous reports of metastatic breast cancer patients treated in the Stockholm Region more than 90% of patients had undergone a metastatic biopsy [43]. However, fine needle aspiration was previously favoured, where only *ERBB2* gene amplification can be assessed and not protein expression. This preference has been abandoned in recent years with the emergence of immunohistochemistry-based predictive biomarkers (Programmed Death Ligand 1, HER2-low). Finally, rescoring of HER2 status was not performed in the entire population, but rather in a small representative sample. Although distinguishing HER2 0 from HER2-low breast cancer has only become relevant in recent years, our results and previous studies have shown that concordance between rescored and original assessments is high [44], limiting thus the potential impact of this limitation.

In conclusion, HER2 status as a predictive biomarker exhibits considerable temporal and spatial variability, as well as long-term prognostic implications. Obtaining tissue for testing if it’s considered safe for the patient is crucial as it increases the likelihood of treatment with trastuzumab deruxtecan which has been shown to improve survival of patients with metastatic HER2-low disease. Its ongoing assessment at the preoperative (Clinicaltrial.gov identifier NCT05113251, NCT05900206) and residual disease settings (NCT04622319) underscores the need for a deep understanding of the long-term implications of HER2 status.

### Supplementary information

Supplementary data

ESMO-GROW checklist

## Data Availability

The dataset that supports the findings of this study is available from the corresponding author upon reasonable request.
