# Supplementary material for: A population-based study on trajectories of HER2 status during neoadjuvant chemotherapy for early breast cancer and metastatic progression
Source: Br J Cancer. 2024 Jun 28;131(4):718–28. doi: 10.1038/s41416-024-02777-6 (PMC11333620; doi:10.1038/s41416-024-02777-6)

Data Supplement to

A population-based study on trajectories of HER2 status during neoadjuvant chemotherapy for early breast cancer and metastatic progression

Caroline Boman, Xingrong Liu, Louise Eriksson Bergman, Wenwen Sun, Christian Tranchell, Maria Angeliki Toli, Balazs Acs, Jonas Bergh, Theodoros Foukakis, Alexios Matikas

Table of Contents

Table S1. Patient characteristics in the ER-positive population **2**
Table S2. Patient characteristics in the ER-negative population **4**
Table S3. Association of neoadjuvant chemotherapy with pCR **6**
Figure S1. Distribution of HER2 status in primary, residual and metastatic biopsies **7**
Figure S2. Distribution of HER2 status across ER expression **8**
Figure S3. HER2 low status according to number of obtained biopsies **9**
Figure S4. Sankey diagram of change of HER2 status from primary to residual disease ... **10**
Figure S5. Overall survival according to HER2 status of primary disease **11**
Figure S6. Adjusted BCFS and DRFS according to HER2 status of primary disease **12**
Figure S7. Survival probabilities according to HER2 status of residual disease **13**
Figure S8. Overall survival according to HER2 loss/gain **14**
Figure S9. Overall survival based on multiple imputation analysis **15**
Figure S10. Distribution of HER2 status according to metastasis site **16**
Figure S11. Overall survival according to HER2 status of metastatic disease **17**

**Supplementary Table 1.** Distribution of patient characteristics according to prechemotherapy HER2 status in the ER-positive population				
	**HER2 0 N (%)**	**HER2-low N (%)**	**HER2-positive N (%)**	**P-value ***
**Patients**	334	662	491	
**Median age (IQR)**	50.9 (44.0-58.9)	51.2 (43.8-61.8)	51.2 (42.5-61.0)	0.506
**Chemotherapy**				<0.001
Antracycline and taxane	273 (81.7)	526 (79.5)	375 (76.4)	
Antracycline	20 (6.0)	31 (4.7)	5 (1.0)	
Taxane	41 (12.3)	104 (15.7)	82 (16.7)	
Other	0 (0.0)	1 (0.2)	29 (5.9)	
**T stage**				0.008
T0-1	51 (15.3)	98 (14.9)	65 (13.2)	
T2	186 (55.9)	357 (54.2)	319 (65.0)	
T3	80 (24.0)	175 (26.6)	96 (19.6)	
T4	16 (4.8)	29 (4.4)	11 (2.2)	
Missing	1	3	0	
**Nodal status**				<0.001
Negative	141 (42.5)	264 (39.9)	251 (51.1)	
Positive	191 (57.5)	397 (60.1)	240 (48.9)	
Missing	2	1	0	
**Histologic type**				
Ductal	226 (72.0)	493 (77.6)	413 (88.4)	<0.001
Lobular	44 (14.0)	59 (9.3)	11 (2.4)	
Other	44 (14.0)	83 (13.1)	42 (9.2)	
Missing	20	27	24	
**Grade**				<0.001
Grade 1	14 (5.0)	28 (4.7)	4 (0.9)	
Grade 2	166 (59.1)	362 (60.7)	207 (46.7)	
Grade 3	101 (35.9)	206 (34.6)	232 (52.4)	
Missing	53	66	48	
**Progesterone receptor**				0.029
Negative	96 (28.7)	172 (26.0)	163 (33.2)	
Positive	238 (71.3)	489 (74.0)	328 (66.8)	
Missing	0	1	0	
**Ki67**				<0.001
Median (IQR)	32 (20-50)	32 (22-50)	40 (28- 51)	
Missing	4	23	12	
**Year of diagnosis**				<0.001
2007-2010	67 (20.1)	122 (18.4)	41 (8.4)	
2011-2014	80 (24.0)	144 (21.8)	101 (20.6)	

2015-2018	118 (35.3)	234 (35.3)	239 (48.7)	
2019-2020	69 (20.7)	162 (24.5)	110 (22.4)	

* Kruskal Wallis tests for continuous variables (age, Ki67); Chi-square tests used for all others.
Abbreviations: IQR: interquartile range

**Supplementary Table 2.** Distribution of patient characteristics according to prechemotherapy HER2 status in the ER-negative population				
	**HER2 0 N (%)**	**HER2-low N (%)**	**HER2-positive N (%)**	**P-value ***
**Patients**	308	184	326	
**Median age (IQR)**	49.0 (40.8-60.4)	53.0 (44.4-65.6)	53.7 (45.6-62.4)	0.003
**Chemotherapy**				<0.001
Antracycline and taxane	279 (90.6)	161 (87.5)	230 (70.6)	
Antracycline	20 (6.5)	13 (7.1)	3 (0.9)	
Taxane	9 (2.9)	9 (4.9)	62 (19.0)	
Other	0 (0.0)	1 (0.5)	31 (9.5)	
**T stage**				0.035
T0-1	59 (19.2)	36 (19.6)	53 (16.3)	
T2	198 (64.5)	111 (60.3)	181 (55.7)	
T3	41 (13.4)	29 (15.8)	71 (21.8)	
T4	9 (2.9)	8 (4.3)	20 (6.2)	
Missing	1	0	1	
**Nodal status**				<0.001
Negative	166 (54.6)	91 (50.0)	126 (38.8)	
Positive	138 (45.4)	91 (50.0)	199 (61.2)	
Missing	4	2	1	
**Histologic type**				0.020
Ductal	249 (83.8)	147 (86.5)	276 (91.1)	
Lobular	2 (0.7)	1 (0.6)	5 (1.7)	
Other	46 (15.5)	22 (12.9)	22 (7.3)	
Missing	11	14	23	
**Grade**				0.043
Grade 1	1 (0.4)	0 (0.0)	1 (0.3)	
Grade 2	45 (16.0)	33 (19.1)	76 (26.0)	
Grade 3	236 (83.7)	140 (80.9)	215 (73.6)	
Missing	26	11	34	
**Progesterone receptor**				0.891
Negative	301 (97.7)	180 (97.8)	317 (97.2)	
Positive	7 (2.3)	4 (2.2)	9 (2.8)	
Missing				
**Ki67**				<0.001
Median (IQR)	70 (50-80)	65.0 (40.0, 80.0)	50 (33-65)	
Missing	2	2	5	
**Year of diagnosis**				0.020
2007-2010	29 (9.4)	24 (13.0)	62 (19.0)	
2011-2014	57 (18.5)	37 (20.1)	69 (21.2)	
2015-2018	125 (40.6)	73 (39.7)	112 (34.4)	

2019-2020	97 (31.5)	50 (27.2)	83 (25.5)	

* Kruskal Wallis tests for continuous variables (age, Ki67); Chi-square tests or exact tests used for all others.
Abbreviations: IQR: interquartile range

Supplementary Table 3. Univariate and multivariable logistic regression for the endpoint of pathologic complete response, according to administered neoadjuvant chemotherapy and per HER2 status.

	HER2 0		HER2-low	
**Treatment**	Crude OR* (95% CI)	Adjusted OR** (95% CI)	Crude OR* (95% CI)	Adjusted OR** (95% CI)
**Anthracycline and taxane**	1	1	1	1
**Anthracycline**	0.38 (0.13, 1.08)	0.42 (0.14, 1.28)	0.68 (0.26, 1.77)	0.66 (0.23, 1.87)
**Taxane**	0.15 (0.03, 0.61)	0.26 (0.06, 1.13)	0.40 (0.19, 0.85)	0.80 (0.36, 1.80)

*Univariate regression analysis
**Multivariable analysis, including covariates: age at diagnosis, ER status, T stage, nodal status, Ki67.
Abbreviations: OR: odds ratio; CI: Confidence Intervals

Supplementary Figure 1. Distribution of HER2-positive, HER2-low and HER2 0 in total unmatched samples at baseline, residual disease and at metastasis (A), in ER+ samples (B) and in ER- samples (C). Distribution of HER2-positive, HER2-low and HER2 0 in matched samples at baseline, residual disease and at metastasis for patients with available data from all three timepoints (D), in ER+ samples (E) and in ER- samples (F).

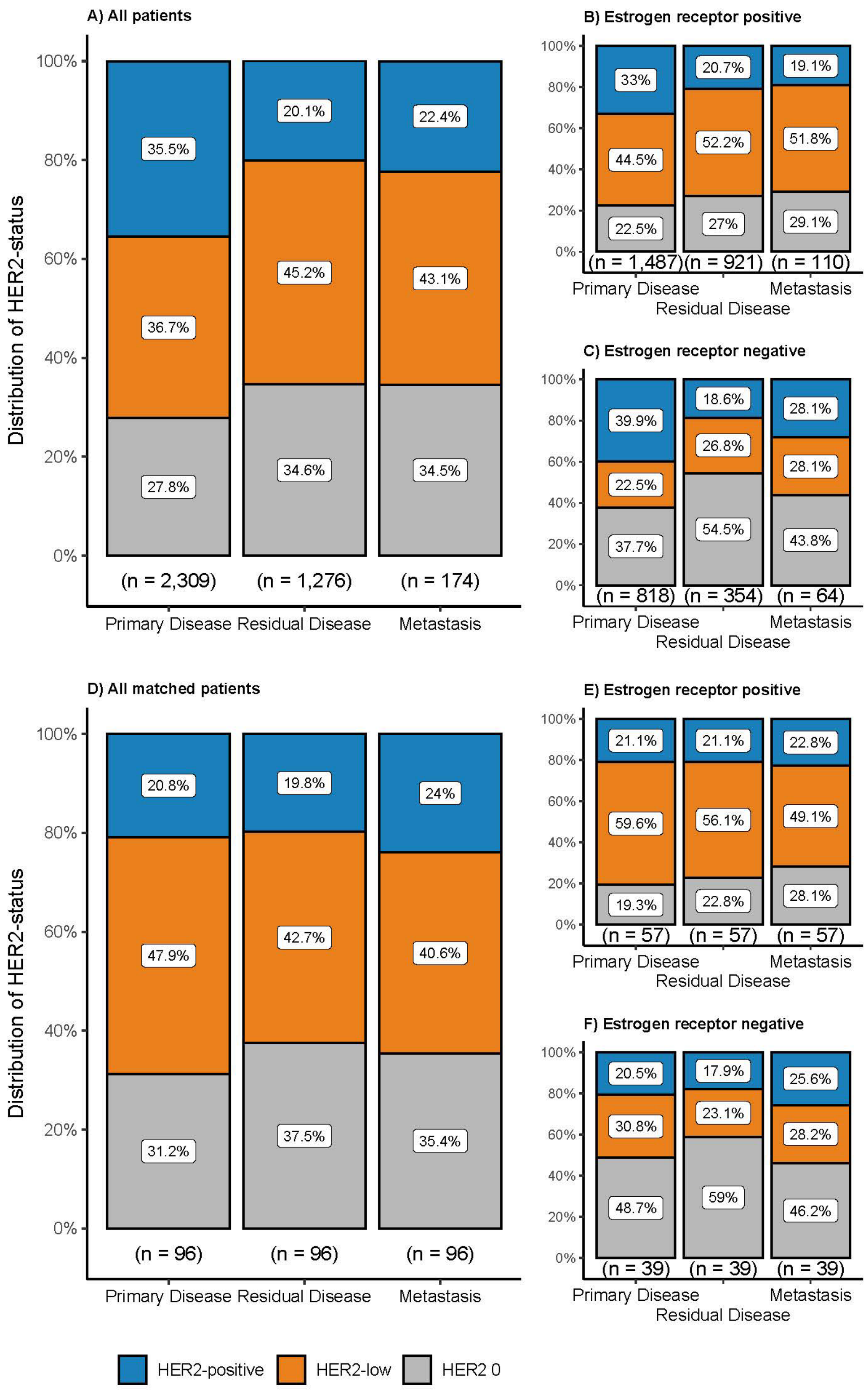

Supplementary Figure 2. Distribution of HER2 status across ER expression. A: negative/positive; B: HER2 0/low

A

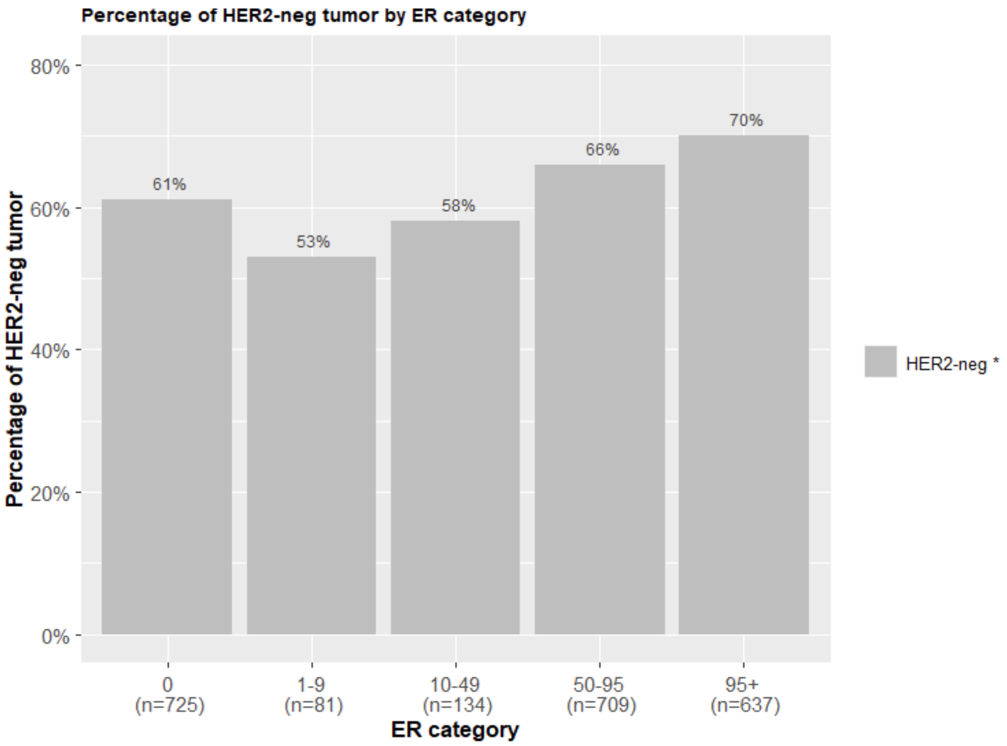

B

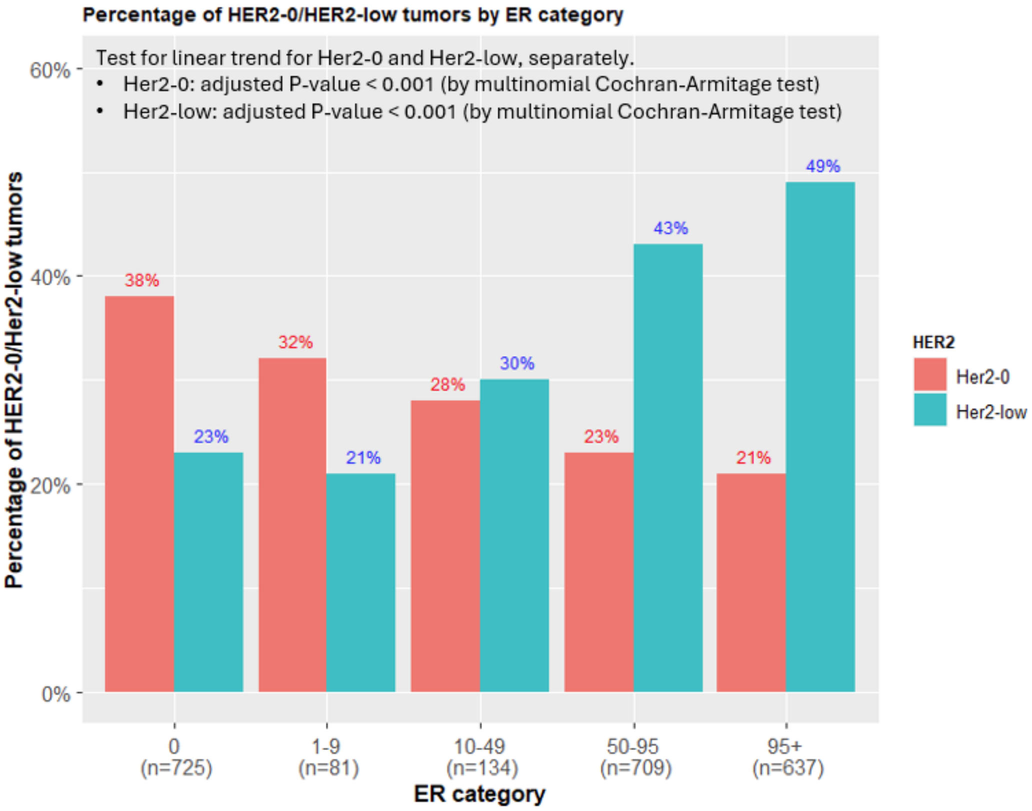

Supplementary Figure 3. Probability of HER2 low status according to number of obtained biopsies at any disease setting in A) estrogen receptor (ER) negative and B) ER-positive disease.

A

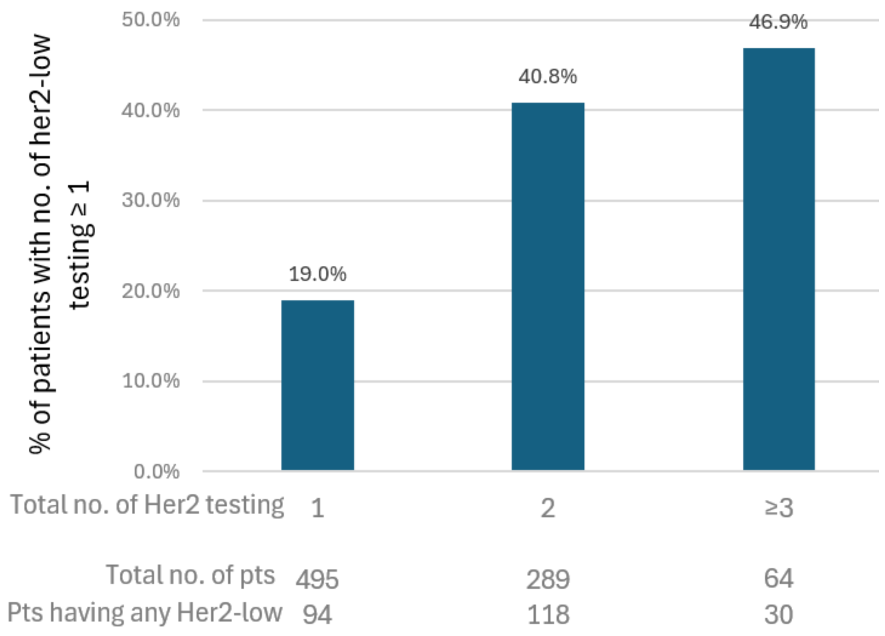

B

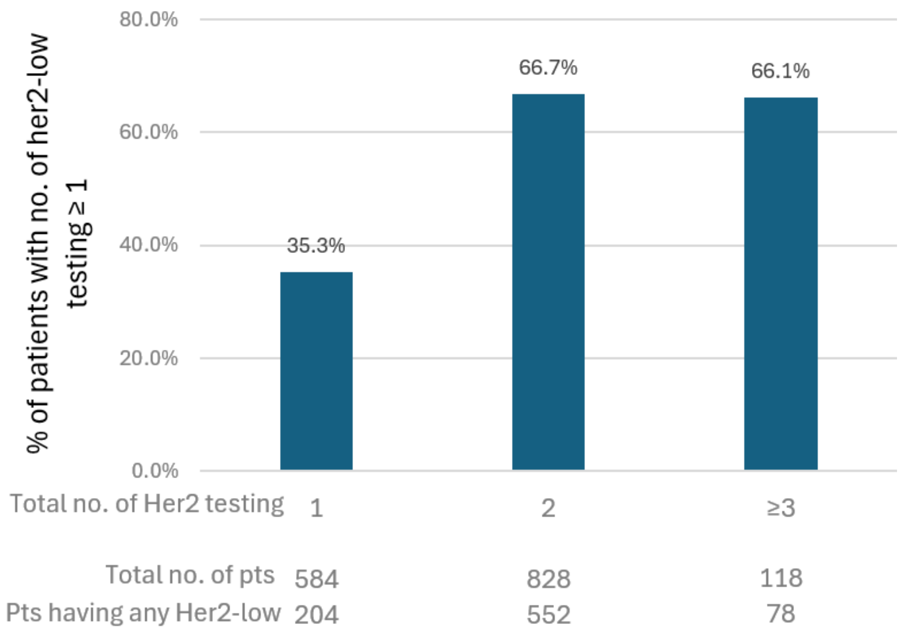

Supplementary Figure 4. Sankey diagram of change of HER2 status from primary to residual disease following neoadjuvant chemotherapy, including patients achieving pathologic complete response and those with missing information on HER2 status

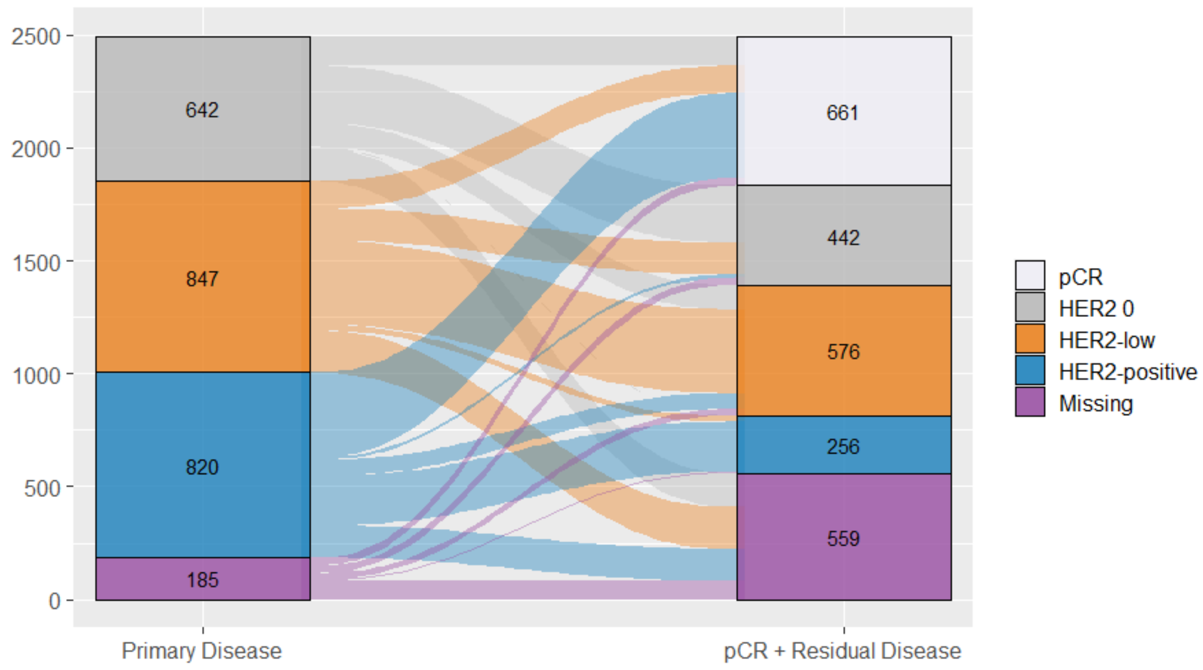

Supplementary Figure 5. Kaplan-Meier curves for overall survival according to HER2 status of primary disease

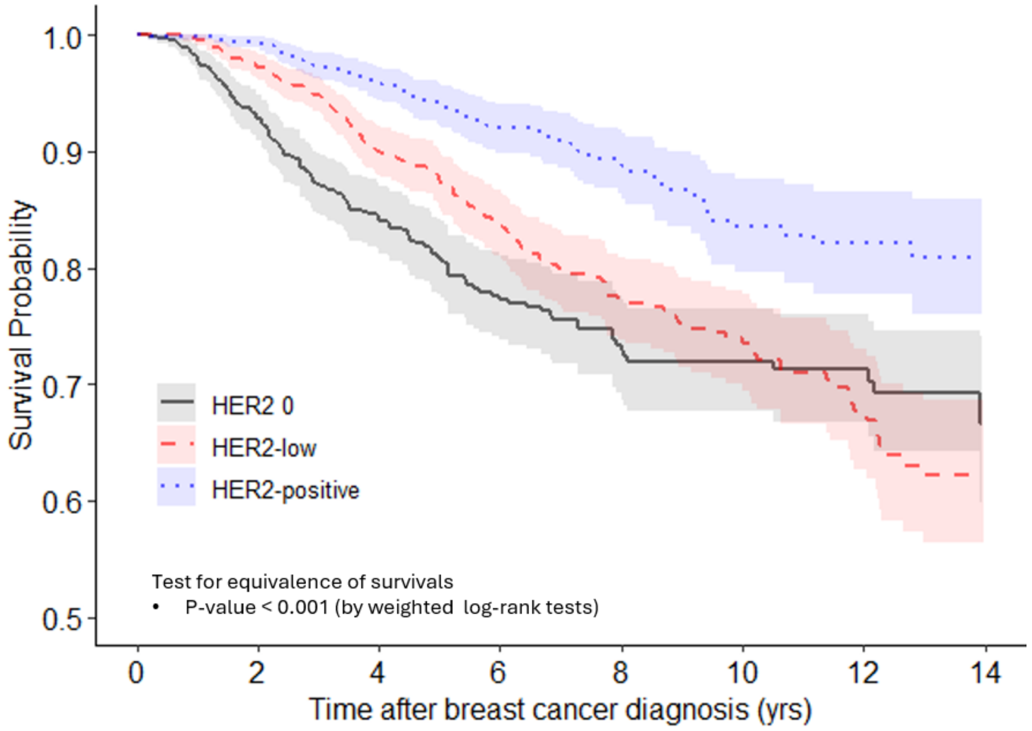

Supplementary Figure 6. Survival probabilities according to HER2 status of primary disease, adjusted for age, chemotherapy, nodal status, T stage, grade, ER status and Ki67. A) Breast cancer free survival. B) Distant relapse free survival.

A

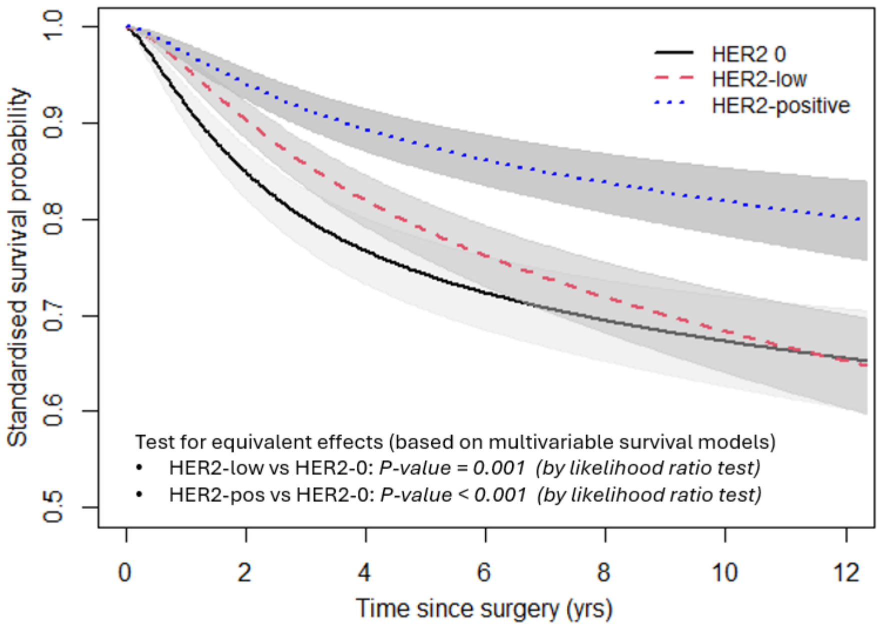

B

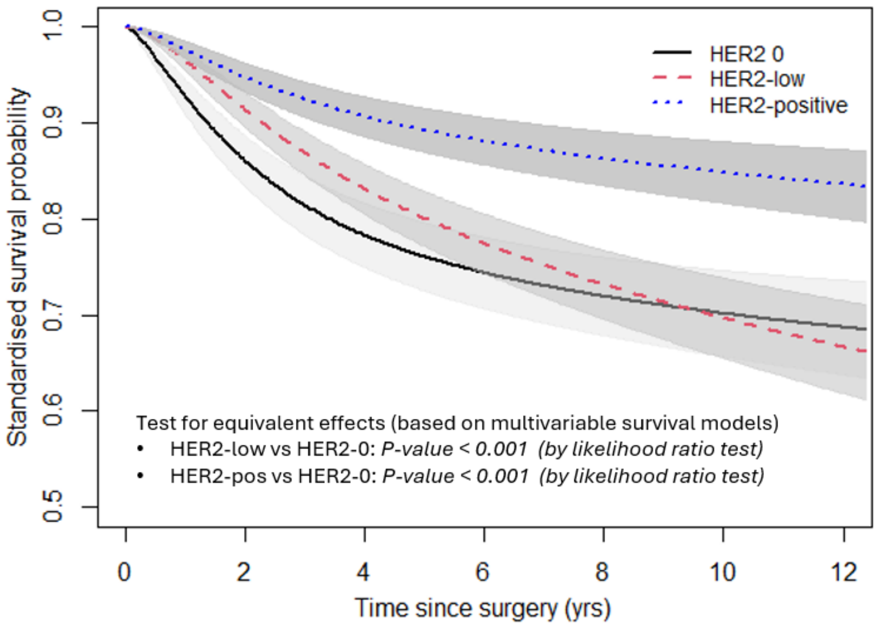

Supplementary Figure 7. Survival probabilities according to HER2 status of residual disease, adjusted for age, chemotherapy, nodal status, T stage, ER status and Ki67. A) Breast cancer free survival. B) Distant relapse free survival. C) Overall survival

A

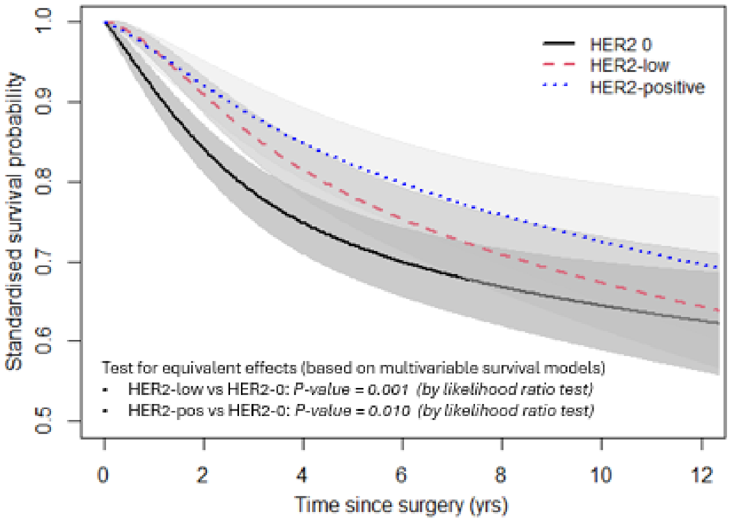

B

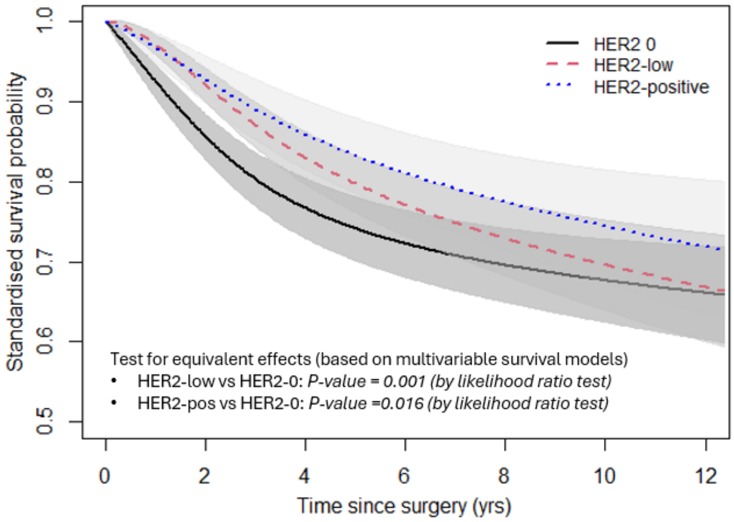

C

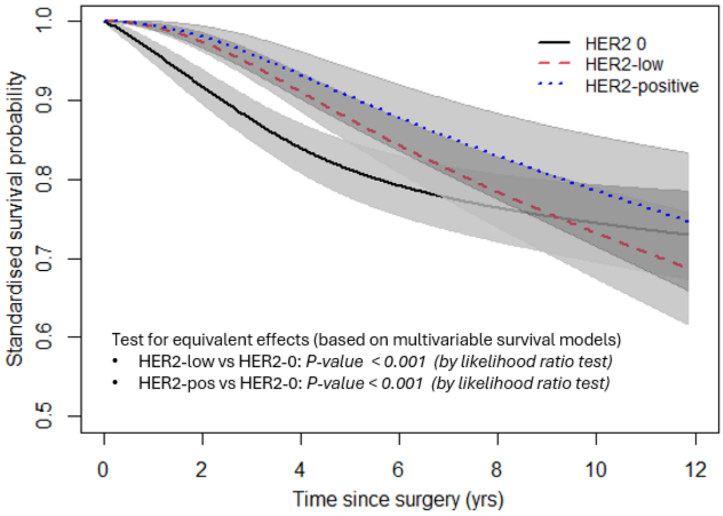

Supplementary Figure 8. Comparison of overall survival estimates between change from HER2 negative status in primary disease to HER2 positive in residual disease (HER2 gain; A) or vice versa (HER2 loss; B) with stable HER2 status during neoadjuvant chemotherapy.

A

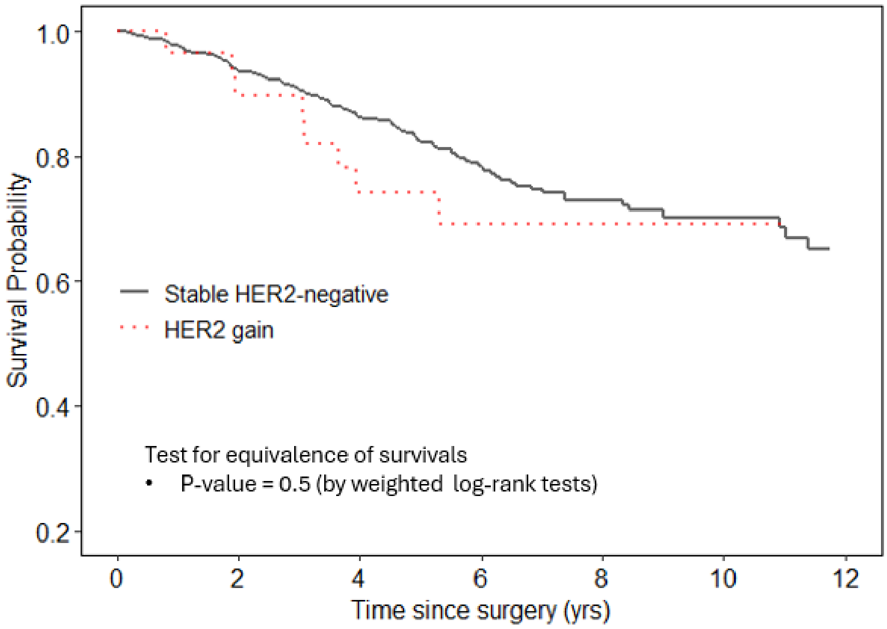

B

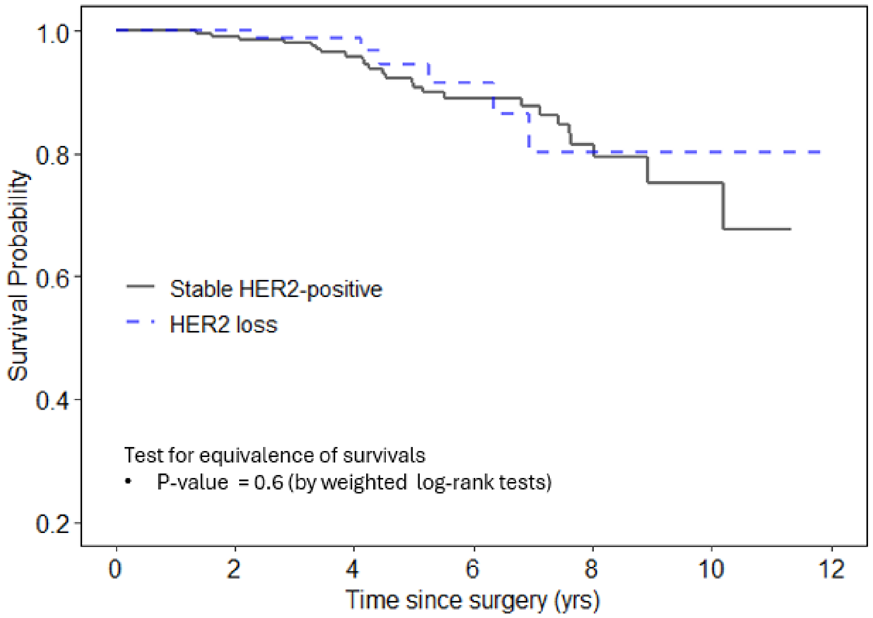

Supplementary Figure 9. Comparison of overall survival estimates according to HER2 status of primary disease between multiple imputation for missing data (500 imputed datasets) and complete case analysis of all patients with available information.

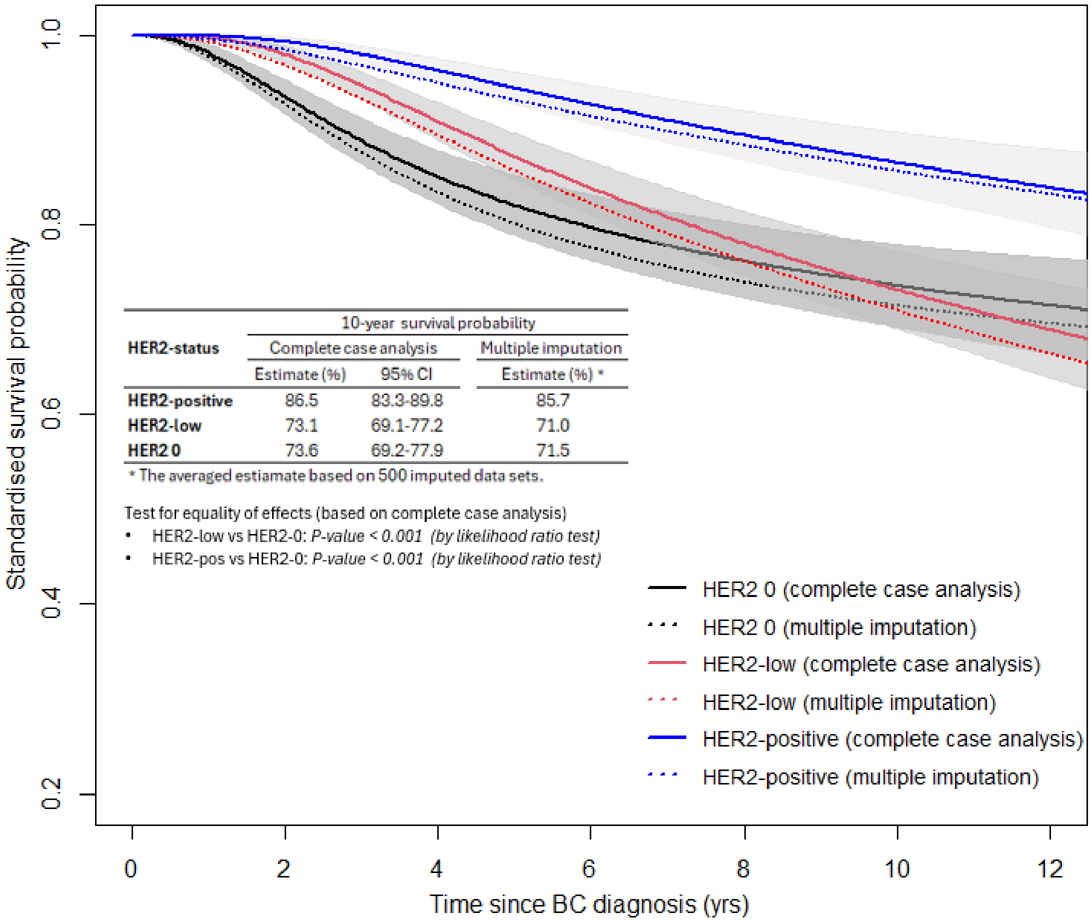

Supplementary Figure 10. Distribution of HER2 status according to metastasis site for overall population (A), ER-negative (B) and ER-positive (C) breast cancer.

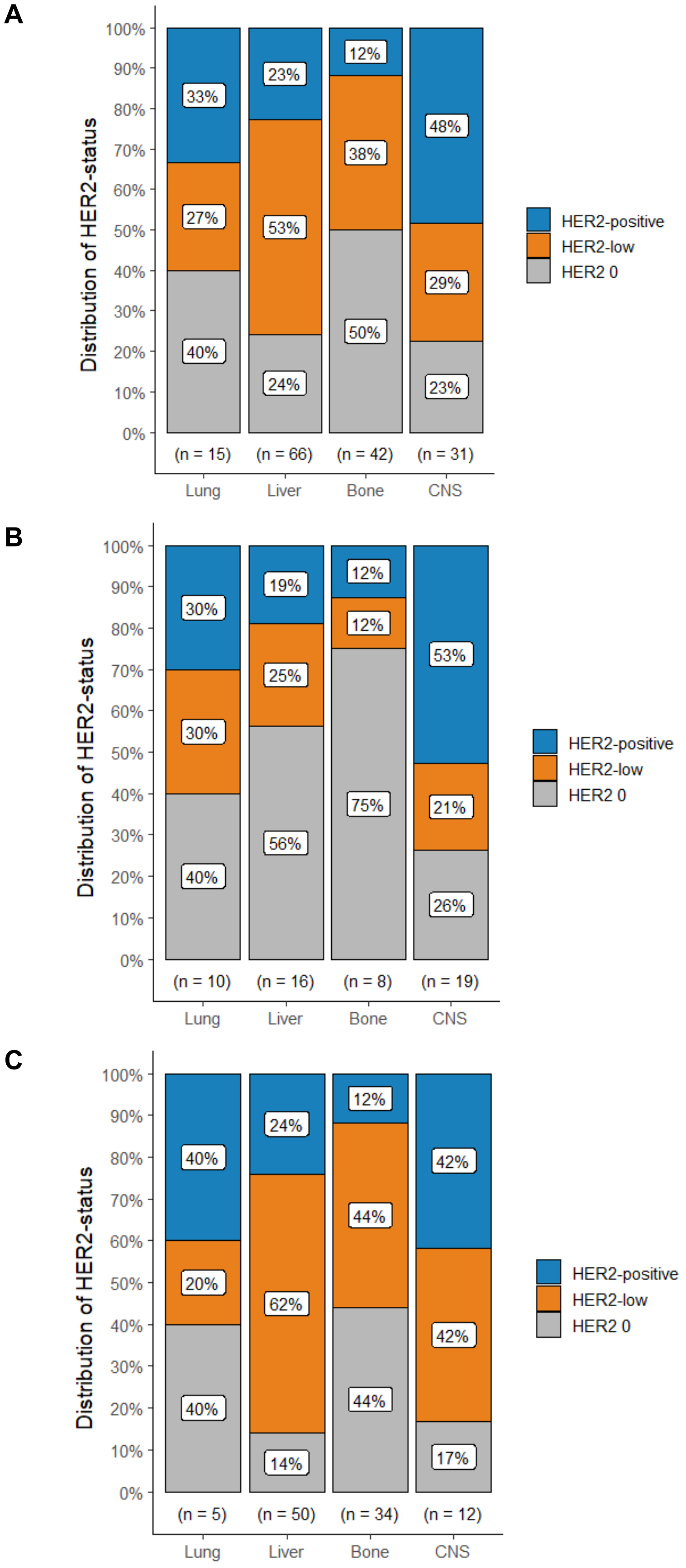

Supplementary Figure 11. A) Kaplan-Meier curves and B) adjusted probability for overall survival according to HER2 status of metastatic disease

A

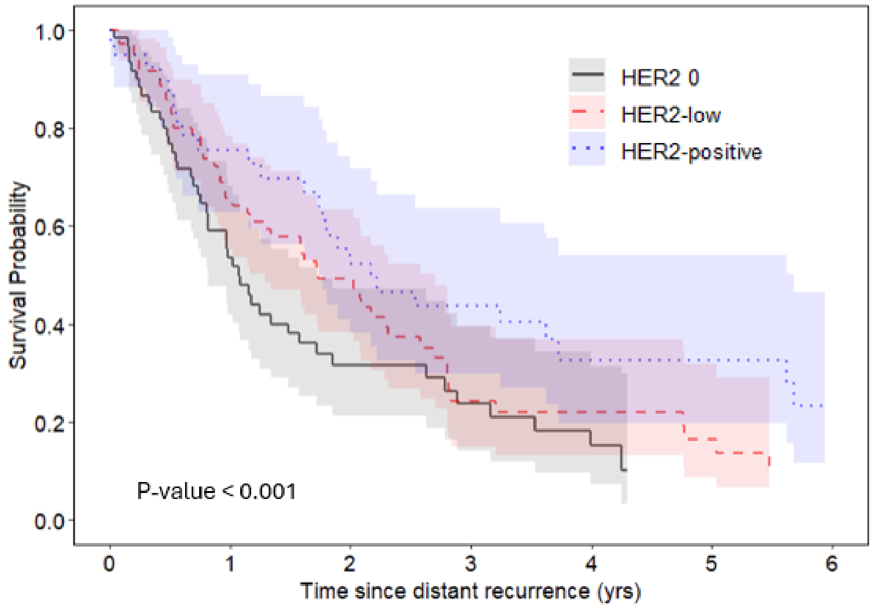

B

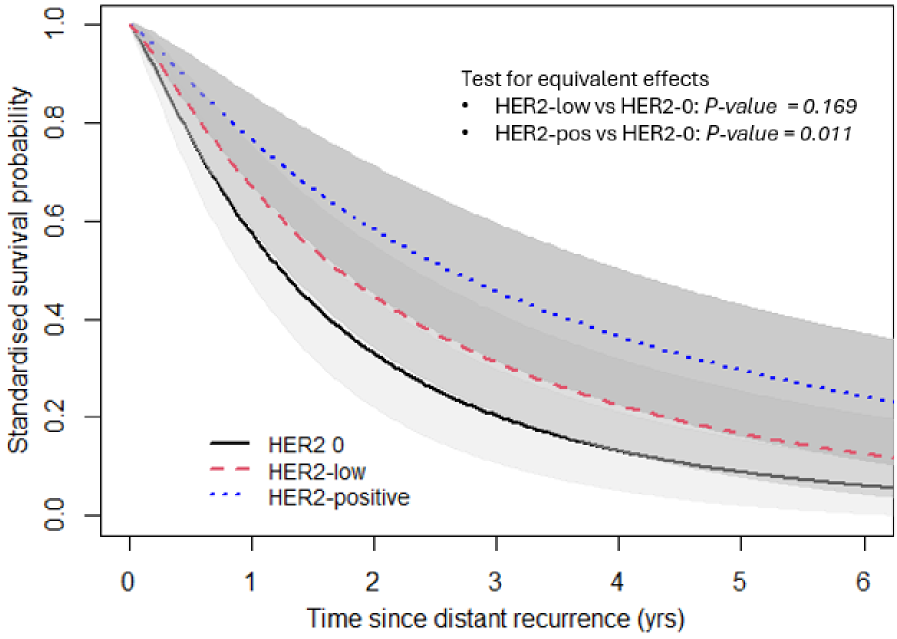

Supplement: Supplementary file 1 — Supplementary data [file 41416_2024_2777_MOESM1_ESM.pdf]
