# Supplementary material for: A population-based study on trajectories of HER2 status during neoadjuvant chemotherapy for early breast cancer and metastatic progression
Source: Br J Cancer. 2024 Jun 28;131(4):718–28. doi: 10.1038/s41416-024-02777-6 (PMC11333620; doi:10.1038/s41416-024-02777-6)
Supplement: Supplementary file 2 — ESMO-GROW checklist [file 41416_2024_2777_MOESM2_ESM.pdf]

ESMO GROW

GUIDANCE FOR REPORTING ONCOLOGY REAL-WORLD EVIDENCE

ESMO-GROW Checklist for Authors and Reviewers

This checklist integrates all ESMO-GROW recommendation and could be used by authors and reviewers when assessing the reporting standards of a real-world evidence study in Oncology.

For the the ESMO-GROW checklist, the following criteria are considered:
"Yes, fully reported" – The recommendation is adequately considered.
"Yes, partially reported" – The recommendation is considered, but some important details are missing.
"Not reported" – The recommendation is applicable for the case, but it was not considered.
"Not applicable" – The recommendation is not applicable for this study.

Name of Author/Reviewer: Alexios Matikas, corresponding author **Date:** 12th March 2024

Title of Manuscript or Identifier: A population based study on trajectories of HER2 status during neoadjuvant chemotherapy for early breast cancer and metastatic progression

Recommendations	Yes, fully reported	Yes, partially reported	Not reported	Not applicable
**1. Title**				
1.1: Concisely include relevant key terms referring to the study type, study population, objectives, data sources and outcomes, depending on the study. Consider including the terms 'real-world' or 'observational'	●	○	○	○
**2. Introduction**				
2.1: Explain the scientific rationale for the research question(s), providing concise background information on previous core evidence from systematic reviews, meta-analyses, clinical trials and/or real-world evidence studies	●	○	○	○
2.2: Identify the gaps in evidence and explain why and how they can be suitably addressed by real-world evidence research. Specify the new evidence that is expected from the current study	●	○	○	○
2.3: Briefly introduce the aim(s) of the study	●	○	○	○
**3. Methods**				
Study objective(s), design, data sources and variables				
3.1: Provide the study research question(s) including a description of the patients or the object under study and the target outcome(s)	●	○	○	○
3.2: Provide the study objective(s) and consider classifying the type of research as descriptive and/or analytical (explanatory or predictive)	●	○	○	○
3.3: Provide relevant information to describe and classify the study design used to address the research question	●	○	○	○
3.4: Give a clear definition of the eligibility criteria used to select the patients or objects under study, particularly regarding cancer-related aspects	●	○	○	○
3.5: Report the specific type and purpose of real-world data source(s) used, providing a detailed description and the reason(s) why the source was considered appropriate for the study objectives	○	●	○	○
3.6: When multiple real-world data sources are used, provide details on interoperability, including identification of duplicated cases or data linkage from separate databases	●	○	○	○
3.7: Provide details and timings of source and study data management. Consider specifying methods of raw data collection, updates and completeness, data extraction, cleaning and/or quality controls and validation	●	○	○	○
3.8: Provide core details on database and/or study registration, governance, ownership, metadata and full data accessibility in the main text or supplementary material	○	●	○	○
3.9: Identify the data source of each core variable, its definition, if the variable was derived or coded, and describe how the derivation or coding was conducted and validated.	○	●	○	○
3.10: Specify the time points of core variables in relation to the cancer disease trajectory	●	○	○	○
3.11: Provide a complete list of core variables included in the study. Variables can be grouped as baseline characteristics, exposure and outcomes or endpoints	○	●	○	○
3.12: For biomarker-related studies, provide details on biomarker description, timing, and methods of assessment and analytical validation	○	●	○	○
Statistical analysis and artificial intelligence methods				
3.13: Summarise the main aspects of the statistical analysis	●	○	○	○
3.14: When applicable, provide details on the pre-planned sample size requirements and power of the study	○	○	○	●
3.15: Specify the pre-planned strategies to identify and mitigate the main sources of bias	○	●	○	○
3.16: Clearly distinguish prespecified from *post hoc* analyses, especially for subgroup analyses	○	●	○	○
3.17: Provide information on internal and external validity, as well as any sensitivity analyses	●	○	○	○
3.18: For analytical studies, the full version of the statistical analysis plan should be provided in the supplementary material, including a brief explanation of any amendments	○	○	●	○
3.19: When applicable, specify which machine learning, deep learning or alternative artificial intelligence method has been used	○	○	○	●
3.20: When reporting real-world data analysis with artificial intelligence (e.g. machine learning and deep learning) algorithms, include comprehensive aspects on data pre-processing techniques, feature engineering strategies and model development	○	○	○	●
3.21: Address the artificial intelligence model explainability and interpretability, and present the plan for integration into clinical practice, if applicable	○	○	○	●
3.22: When applicable, briefly describe the multidisciplinary team required for the study and explain how these needs were met	○	○	○	●

ESMO GROW

GUIDANCE FOR REPORTING ONCOLOGY REAL-WORLD EVIDENCE

ESMO-GROW Checklist for Authors and Reviewers

Recommendations	Yes, fully reported	Yes, partially reported	Not reported	Not applicable
**4. Results**				
4.1: Provide number of cases excluded or nonparticipating and reasons at each stage of sample selection, as well as numbers lost to follow-up. Compare the cases excluded with those included in the analyses. Illustrate this with a flowchart	●	○	○	○
4.2: Describe the baseline characteristics of the cases included (e.g. clinico-demographic and tumour characteristics). The baseline characteristics of different groups under analysis should be compared, if applicable	●	○	○	○
4.3: Report the results of the primary analysis of study outcomes. Briefly describe the results of exploratory analyses if relevant (prespecified and/or *post hoc*). Provide details of how readers can access the full results	●	○	○	○
**5. Discussion and conclusions**				
Discussion				
5.1: Summarise the core results that address the primary research question(s) and objectively discuss the data in relation to the best available evidence on the topic. Avoid a convenient selection of literature to support a point	●	○	○	○
5.2: Discuss the strengths and limitations of the current study, including the main biases, how the strategies applied contributed to bias avoidance or mitigation, and, if applicable, in which direction the authors estimate that residual bias may influence the core results of the study	●	○	○	○
5.3: Discuss the generalisability of the study results and their potential implications for clinical practice, health policies or public health and for the generation of hypotheses for future research	●	○	○	○
Conclusions				
5.4: Provide a balanced summary of core results relating to the primary research question and the main implications for clinical practice, health policies and/or public health. Suggest further research considering the remaining unmet needs and limitations from the reported study	●	○	○	○
**6. Final considerations**				
6.1: Specify all relevant study sponsorship(s) as well as direct and/or indirect or in-kind funding	●	○	○	○
6.2: Specify all relevant acknowledgements, author disclosures, individual contributions and other final considerations as per journal regulations	●	○	○	○

Notes:

To access full manuscripts and for citations, please consider the following references and links:

- Castelo-Branco L et al. “ESMO Guidance for Reporting Oncology real-World evidence (GROW)". Ann Oncol 2023; 34: 10.1016/j.annonc.2023.10.001
- Castelo-Branco L et al. “ESMO Guidance for Reporting Oncology real-World evidence (GROW)". ESMO Real World Data & Digital Oncol 2023; 1: 10.1016/j.esmorw.2023.10.001

ESMO GOOD SCIENCE BETTER MEDICINE BEST PRACTICE
